# The path to hepatitis C elimination: who are we leaving behind and why?

**DOI:** 10.1002/jia2.26136

**Published:** 2023-07-26

**Authors:** Jurgen K. Rockstroh, Tracy Swan, Judy Chang, Fauzi Elamouri, Andrew R. Lloyd

**Affiliations:** ^1^ Department of Medicine I University Hospital Bonn Bonn Germany; ^2^ GlobalHealth Consultant Barcelona Spain; ^3^ INPUD London UK; ^4^ Medical School Hamburg Hamburg Germany; ^5^ Kirby Institute Sydney New South Wales Australia

1

The 2016 World Health Organization (WHO) 2030 global elimination targets for hepatitis C virus (HCV) are: 80% of those with chronic HCV treated, 90% reduction in incidence and 65% reduction in liver‐related mortality [1]. These targets have recently been updated in their 2022–2030 Global Health Sector Strategies to an incidence reduction to 5 per 100,000 and mortality reduction to 2 per 100,000 [2]. Other than in a few countries (e.g. Egypt), elimination targets are endangered in the majority of the world—regardless of income level [3]. This is largely because the greater burden of HCV infection is carried by marginalized populations of people who use drugs (PWUD) and people who are incarcerated, who are being left behind in HCV control efforts.

Biomedical advances—such as the advent of direct‐acting antiviral therapies, with cure rates above 95%, and simplified screening via rapid point‐of‐care tests—are key components for the road to elimination. The remaining major challenges are in resourcing and implementation, particularly for vulnerable populations. PWUD and people who are incarcerated carry the largest burden of HCV infection, and are being left behind in elimination efforts [4].

So far, around 30% of the 2.3 million people with chronic HCV in the United States have been treated, whereas only 10% of PWUD who are living with HCV have been treated. Unfortunately, in several states and countries without socialized healthcare systems, some insurance payers require abstinence from substance use to be eligible for access to HCV services, thereby creating additional access barriers for PWUD—despite the lack of data supporting this restriction. The scarcity of comprehensive harm reduction, criminalization of drug use and the persistent stigmatization of PWUD remain as important barriers to HCV elimination [5]. There is a growing body of evidence indicating that PWUD and people who are incarcerated can be effectively engaged in HCV treatment with successful outcomes, particularly when care is co‐delivered with harm reduction approaches [6, 7]. Nevertheless, in high‐income countries, HCV treatment rates remain much lower among PWUD, due to a lack of simplified HCV testing and scale‐up of harm reduction‐based HCV treatment programmes. PWUD face numerous additional barriers to HCV services, including limited transportation, poor geographic access to speciality care, social stigma and discrimination, homelessness, and negative clinicians’ attitudes towards treating PWUD [8]. Recent data from the United States suggest that the failure to provide HCV care and treatment to PWUD can lead to 20 unique infections for each person left untreated—in just 3 years [9]. This underlines the importance of accessible HCV treatment for PWUD, both to improve their individual health and as a public health measure.

For HCV treatment‐as‐prevention (TasP) to be effective, people who inject drugs, who are at the highest risk for ongoing transmission, must be actively engaged. However, they often have limited and unpleasant interactions with the healthcare system. Clearly, this calls for different models of care which are easier to access, without classical hierarchical structures, and ideally combine social and other services (e.g. meals, hygiene products, clean syringes and needles) along with HCV testing and treatment. Having all these services under one roof will help to retain people in care, and increase treatment uptake. A meta‐analysis reviewing primary care and community‐centred HCV treatment indicated that these innovative models could improve uptake and completion of treatment, while maintaining comparable cure rates, regardless of ongoing drug use [10]. Moreover, qualitative interviews of methadone maintenance programme beneficiaries suggest that treatment with methadone or buprenorphine can reduce the frequency of injecting and HCV‐related feelings of shame, and improve their self‐care. At the height of the COVID‐19 pandemic, pioneering telehealth programmes successfully delivered HCV care and treatment to people receiving opioid agonist therapy [11].

HCV care in correctional facilities is generally even more inaccessible than in the community [12]. Further, instead of maximizing the opportunity for testing, treatment and cure, prisons are recognized to be a key venue for ongoing transmission [13]. Although HCV treatment in custodial settings is feasible, and comes with the usual high HCV cure rates, globally, only 23 countries include HCV services for incarcerated people in their national hepatitis plans [14]. A recent landmark study demonstrated that scale‐up of treatment in the Australian prison setting reduced the incidence of HCV transmission—that is, TasP [15]. Importantly, the study also revealed a high rate of re‐infection, despite reasonable opioid agonist therapy coverage, highlighting the need for improved prevention measures—including increased opioid agonist therapy coverage and prison needle‐syringe programmes.

By contrast, in Germany, testing and treatment of HCV within prisons is regarded as a federal jurisdictional responsibility, and so ends up being mostly left to the authorities running the individual prisons. This means that in some prisons, HCV screening and treatment are implemented, but in many others, neither takes place. These contrasts in high‐income countries are even more stark in most low‐ and middle‐income countries, where very few prison‐based HCV or harm reduction services for PWUD exist. There is a clear argument for international benchmarking for these services in the prison sector, based on the fundamental premise of the United Nations Nelson Mandela Rules, which emphasize that the provision of healthcare for prisoners is a state responsibility and should be governed by the same ethical and professional standards as those applicable to patients in the community [16]. Given the importance of prison‐based HCV testing and treatment for national and global elimination efforts, the lack of prison‐specific impact and coverage targets in the WHO guidance is a significant gap in public health policy.

In summary, PWUD and prisoners, two vulnerable populations with considerable overlap who continue to experience a high prevalence of chronic HCV, are currently left behind in the HCV elimination pathway—threatening to stagnate or reverse progress towards the 2030 elimination targets. This threat reflects the fact that society remains far from driving the political will to intervene in these populations, far from successful de‐stigmatization, and far from the ability to collectively uphold the human rights of PWUD and those who are incarcerated. There is a clear need for changes in punitive laws and policies which drive people away from services, generate mistrust and undermine funding allocations.

## COMPETING INTERESTS

JKR has received Honoraria for consulting or speaking at educational events from AbbVie, Boehringer, Gilead Sciences, Merck, Janssen and ViiV. ARL has received investigator‐initiated research grant support from Gilead Sciences and AbbVie. TS, JC and FE have no competing interests to report.

## AUTHORS’ CONTRIBUTIONS

JKR, TS, JC, FE and ARL contributed ideas, and drafting and review of the article.
